# Impact of Heat and Humidity on Critical Power and Physiological Responses during the Three-Minute All-Out Test in Cyclists

**DOI:** 10.5114/jhk/219785

**Published:** 2026-04-02

**Authors:** Yu-Hsuan Kuo, Yun-Rong Yang, Huey-June Wu, Yu-Chi Kuo

**Affiliations:** 1 Department of Physical Education, Chinese Culture University, Taipei, Taiwan.; 2 Department of Physical Education and Sports Sciences, National Taiwan Normal University, Taipei, Taiwan.; 3 Graduate Institute of Sport Coaching Science, Chinese Culture University, Taipei, Taiwan.; 4 Department of Exercise and Health Science, National Taipei University of Nursing and Health Sciences, Taipei, Taiwan.

**Keywords:** high-intensity exercise, critical threshold, environmental stress, exercise performance

## Abstract

The purpose of this study was to examine the effects of high ambient temperature and varying humidity levels on critical power (CP) and physiological variables in cyclists. Twelve male cyclists (age 36 ± 8 years, body height 172 ± 4 cm, body mass 72 ± 10 kg) performed incremental exercise tests (IET) and 3-min all-out tests (3MT) under three environmental conditions: high temperature with high relative humidity (Ht-wet), high temperature with low relative humidity (Ht-dry), and neutral temperature with low relative humidity (Nt-dry). Physiological responses, including maximal oxygen uptake (VO_2max_) and ventilatory thresholds (VT_1_, VT_2_) were assessed relative to power output (wVO_2max_, wVT_1_, wVT_2_). End power (EP), anaerobic work capacity (WEP), and time to exhaustion were also measured. Results showed that VO_2max_ and wVO_2max_ did not differ among conditions, whereas VT_2_ and wVT_1_ were highest under the Nt-dry, intermediate under the Ht-dry, and lowest under the Ht-wet condition (p < 0.05). For VT_1_ and wVT_2_, values under the Ht-wet condition were significantly lower than under Nt-dry and Ht-dry conditions (p < 0.05). During the 3MT, EP, peak power, and average power were significantly higher under the Nt-dry than under Ht-dry and Ht-wet conditions (p < 0.05). In conclusion, high temperature reduced 3MT performance compared with the neutral condition, with no additional decline between Ht-wet and Ht-dry conditions. In contrast, VT and wVT were most reduced under the Ht-wet condition, indicating greater impairment of submaximal physiological responses under hot-humid conditions. These results suggest that humidity mainly affects threshold-related outcomes rather than 3MT performance. Environmental heat should therefore be considered when applying 3MT-derived variables for training prescription.

## Introduction

Fatigue is a multifactorial and dynamic process that limits exercise performance when intensity exceeds an individual’s sustainable capacity. This critical exercise intensity has traditionally been termed critical power (CP), which represents the boundary between heavy and severe exercise domains, distinguishing sustainable from unsustainable intensities (Galán-Rioja et al., 2024; Jones and Vanhatalo, 2017; Vanhatalo et al., 2011). Traditionally, CP is determined using multiple time-to-exhaustion trials, which are time-consuming and impractical for applied or large-scale testing (Vinetti et al., 2019).

To address these limitations, the 3-minute all-out test (3MT) was developed as a practical single-bout alternative for estimating CP (Hunter et al., 2023; Sreedhara et al., 2023). The 3MT is especially valued for its practicality, allowing for large-scale or field-based testing without the need for multiple exhaustive sessions (Pettitt et al., 2025). The 3MT has been validated across a variety of exercise modalities, showing strong correlations between EP and CP (Burnley et al., 2006; Vanhatalo et al., 2007). The 3MT has demonstrated good reliability and validity in various exercise modalities under laboratory temperate conditions (Cheng et al., 2012; Kaiser et al., 2021).

However, most validation studies have been conducted in neutral environments, which do not represent the thermal challenges encountered in real-world training and competition. With global warming projected to raise average temperatures by approximately 1.5°C within the next two decades (IPCC, 2021), athletes are increasingly required to train and compete in hot environments. Exercising in the heat imposes significant physiological stress by increasing cardiovascular demand, impairing thermoregulation, and accelerating fatigue (Nybo et al., 2014; Périard et al., 2015). Previous studies have indicated that performance outcomes of the 3MT can be affected by environmental stressors (Kuo et al., 2017). Under hot outdoor conditions (~34.5°C), Kuo et al. (2017) reported that critical velocity showed moderate to high reliability, but values were consistently lower than those obtained in temperate environments. These findings suggest that CP- or EP-derived indices from the 3MT may be environment-dependent, particularly under high thermal stress, which may limit their accuracy for training prescription and performance monitoring.

Relative humidity (RH) further exacerbates thermal stress by impairing evaporative heat loss, leading to greater elevations in core temperature and reductions in endurance capacity (Armstrong et al., 2007; Moyen et al., 2014). Armstrong (2000) proposed that above ~16°C, steady-state core temperature during exercise increases proportionally with RH. More recent work has confirmed that hot-humid conditions impair endurance capacity more severely than hot-dry conditions, even at comparable ambient temperatures (Flouris and Schlader, 2015). These findings suggest that RH must be considered when evaluating aerobic capacity and endurance in hot environments (Baillot et al., 2021; Maughan et al., 2012). While previous studies have shown that the 3MT may be influenced by heat exposure (Kuo et al., 2017), the specific effects of humidity on EP remain unclear.

Given the increasing use of the 3MT in field-based assessments, it is important to determine whether EP remains a valid indicator of aerobic performance under different thermal and humidity conditions. Therefore, the purpose of this study was to investigate the effects of high temperature combined with different humidity levels on EP obtained from the 3MT in recreational cyclists.

## Methods

### Participants

Based on previous studies reporting large effects of environmental temperature and humidity on EP and cycling endurance performance (Baillot et al., 2021; Kuo et al., 2021), an a priori power analysis was conducted using G*Power (version 3.1.9.7). With an alpha level of 0.05, statistical power of 0.80, and an expected effect size of 0.45, the required sample size was estimated to be at least 10 participants. Twelve male cyclists, engaging in >6 h·wk^−1^ of cycling training, volunteered for this study. All participants completed a health questionnaire confirming no history of smoking, pulmonary, cardiovascular, or respiratory disorders. Written informed consent was obtained prior to participation. Subjects abstained from caffeine and alcohol for 48 h, and they fasted for 4 h before testing. All tests were performed at consistent times of the day to minimize circadian variation. The study protocol was approved by the Institutional Review Board of the Fu Jen Catholic University, New Taipei City, Taiwan (protocol code: C110083; approval date: 28 April 2022). The participants had mean VO_2__max_ of approximately 56–58 ml·kg^−1^·min^−1^ across all conditions, indicating a recreationally trained to moderately trained endurance capacity. Descriptive statistics for participants’ baseline characteristics and environmental conditions are presented in Table 1.

**Table 1:** Participants’ baseline data and environmental conditions.

Variables	(Mean ± SD)		
Age (yrs)	36.1	±	8.0
Body height (m)	1.72	±	0.04
Body mass (kg)	72.3	±	9.9
Training experience (yrs)	4.2	±	1.5
Ht-wet			
Temperature (°C)	34.8	±	0.7
RH (%)	74.8	±	3.9
Ht-dry			
Temperature (°C)	34.9	±	0.8
RH (%)	36.0	±	2.9
Nt-dry			
Temperature (°C)	21.9	±	0.6
RH (%)	38.3	±	3.0

Ht-wet: a high temperature with high relative humidity condition; Ht-dry: a high temperature with low relative humidity condition; Nt-dry: neutral temperature with low relative humidity; RH: relative humidity

### Experimental Design

After familiarization with the experimental procedures, each participant performed incremental exercise tests (IET) and 3-minute all-out tests (3MT) in a randomized crossover design under three environmental conditions: high temperature and high humidity (34–36°C, 70–80%RH, Ht-wet), high temperature and low humidity (34–36°C, 30–40%RH, Ht-dry), and neutral temperature and low humidity (21– 23°C, 30–40%RH, Nt-dry). Participants remained seated for 20 min for environmental acclimation before testing (Backx et al., 2000). Trials were separated by ≥72 h. Oxygen uptake and the heart rate (HR) were continuously measured, and ratings of perceived exertion (RPE) were recorded (Borg, 1982) before and immediately after each test.

### Incremental Exercise Test (IET)

The IET protocol (Bailey et al., 2010) consisted of a 3-min warm-up at 0 W, followed by 30 W·min^−1^ increments at 80–90 rpm until exhaustion. Exhaustion was defined by meeting ≥3 of the following (Cheng et al., 2012): (1) respiratory exchange ratio > 1.2; (2) HR ≥ 90% age-predicted maximum (208 − 0.7 × age); (3) VO_2_ plateau (< 150 ml·min^−1^ increase with workload); (4) RPE > 17. Ventilatory thresholds (VT) and VO_2max_ were determined from processed breath-by-breath data. Peak power output was defined as the work rate associated with VO_2max_, VT_1_, and VT_2_ (*w*VO_2max_, *w*VT_1_, and *w*VT_2_).

### Three-Minute All-Out Test (3MT)

The 3MT was conducted on a cycle ergometer according to Vanhatalo et al. (2007). First, VT_1_ and its corresponding power (*w*VT_1_) were identified using the v-slope method (Beaver et al., 1986). Linear resistance for the test was set as *w*VT_1_ + 50% (*w*VO_2max_ − *w*VT_1_). After a warm-up (5 min at 80% *w*VT_1_ with three maximal 10-s sprints), participants rested 20 min before completing the 3MT. Participants mounted the cycle ergometer and performed 3-min unloaded cycling at a self-selected cadence. Five seconds before the start of the test, the cadence was increased to 110 rpm, and the 3MT began upon the command “3, 2, 1, GO”. Participants were instructed to reach maximum speed as quickly as possible and maintain maximal effort throughout the test. End power (EP) was defined as the average power output of the final 30 s, and the total work above EP (WEP). Peak VO_2_ (VO_2peak_) was determined as the highest 10-s average (Vanhatalo et al., 2011).

### Statistical Analysis

Data are presented as mean ± standard deviation (SD). A one-way repeated-measures analysis of variance (ANOVA) was used to examine the effects of environmental conditions on physiological and performance variables obtained from the IET and the 3MT. When a significant main effect was observed (*p* ≤ 0.05), pairwise comparisons were conducted using the least significant difference (LSD) post hoc test. Effect sizes were calculated using Cohen’s *d* and interpreted as small (< 0.50), moderate (0.50–0.79), or large (≥ 0.80) (Cohen, 1988). In addition, 95% confidence intervals (95% CI) were calculated for pairwise differences to provide an estimate of the precision of the observed effects. All statistical analyses were conducted using SPSS Statistics 22.0 (IBM Corp., Armonk, NY, USA).

## Results

### IET Exercise Performance

The effects of environmental conditions on IET performance variables are presented in Table 2. VO_2max_, *w*VO_2max_, and HR_max_ did not differ among conditions. Exercise time to exhaustion showed a significant main effect of the condition (F(2,22) = 3.55, *p* = 0.046). Post hoc analysis indicated that time to exhaustion was significantly reduced under the Ht-dry compared with the Nt-dry condition (*p* < 0.001, *d* = 1.38, 95% CI [16.03, 42.30]). In contrast, it did not differ significantly when comparing Ht-wet and Nt-dry conditions (*p* = 0.086, *d* = 1.22, 95% CI [−4.31, 56.15]).

**Table 2:** Effects of environmental temperature and humidity on IET exercise performance.

Variables	Nt-dry			Ht-dry			Ht-wet			F	*p*
VO_2max_ (ml·kg^−1^·min^−1^)	56.2	±	8.2	57.9	±	9.0	56.9	±	9.1	2.169	0.138
VT_1_ (ml·kg^−1^·min^−1^)	36.3	±	4.5	35.9	±	4.3	32.6	±	4.9^*†^	13.244	0.000
VT_2_ (ml·kg^−1^·min^−1^)	47.5	±	6.8	46.0	±	7.0^*^	43.1	±	5.6^*†^	7.366	0.004
*w*VO_2max_ (W)	315.0	±	37.3	305.0	±	33.4	307.5	±	42.7	1.505	0.244
*w*VT_1_ (W)	187.5	±	26.0	172.5	±	18.7^*^	162.5	±	20.1^*†^	10.450	0.001
*w*VT_2_ (W)	257.5	±	32.5	245.0	±	28.1	227.5	±	29.9^*†^	10.076	0.001
HR_max_ (bpm)	174.6	±	12.1	175.6	±	11.3	173.0	±	11.9	1.982	0.162
Time to exhaustion (s)	810.0	±	74.0	780.8	±	71.6^*^	784.1	±	79.8	3.554	0.046
RPE (score)											
Baseline	6.5	±	0.8	6.7	±	0.8	6.8	±	0.9	0.681	0.516
Post-exercise	17.3	±	1.0	17.8	±	1.1	18.0	±	1.1	3.011	0.070

Note: Ht-wet: a high temperature with high relative humidity condition; Ht-dry: a high temperature with low relative humidity condition; Nt-dry: neutral temperature with low relative humidity; VO_2max_: maximal oxygen uptake; VT_1_: the first ventilatory threshold; VT_2_: the second ventilatory threshold; wVO_2max_: VO_2max_ relative to power output; wVT_1_: VT_1_ relative to power output; wVT_2_: VT_2_ relative to power output; *HR_max_*: maximal heart rate; *RPE*: ratings of perceived exertion; * significantly different from Nt-dry (p < 0.05); † significantly different from Ht-dry (p < 0.05)

For ventilatory thresholds, a significant main effect of the environmental condition was observed for VT_1_ (F(2,22) = 13.24, *p* < 0.001). Post hoc analysis indicated that VT_1_ was significantly lower under the Ht-wet condition compared with both Nt-dry (*p* < 0.001, *d* = 0.72, 95% CI [2.14, 5.43]) and Ht-dry (*p* < 0.001, *d* = 0.63, 95% CI [1.98, 4.61]) conditions. In contrast, no significant difference was observed between Nt-dry and Ht-dry conditions. Similarly, a significant main effect of the environmental condition was observed for VT_2_ (F(2,22) = 7.37, *p* = 0.006). Post hoc analysis showed that VT_2_ was significantly lower under the Ht-wet compared with Nt-dry (*p* = 0.006, *d* = 0.70, 95% CI [1.57, 7.15]) and Ht-dry (*p* = 0.010, *d* = 0.47, 95% CI [0.86, 4.65]) conditions. No significant difference was found between Nt-dry and Ht-dry conditions (*p* = 0.267, *d* = 0.67, 95% CI [−1.28, 4.19]).

For the work-related variables, a significant main effect of the environmental condition was observed for *w*VT_1_ (F(2,22) = 10.45, *p* = 0.001). Post hoc analysis indicated that *w*VT_1_ under the Nt-dry condition was significantly higher than under Ht-dry (*p* = 0.026, *d* = 1.32, 95% CI [2.15, 27.85]) and Ht-wet (*p* = 0.002, *d* = 2.20, 95% CI [11.32, 38.68]) conditions. In addition, *w*VT_1_ under the Ht-dry condition was significantly higher than under the Ht-wet condition (*p* = 0.039, *d* = 0.88, 95% CI [0.62, 19.39]). Similarly, a significant main effect of the environmental condition was observed for *w*VT_2_ (F(2,22) = 10.08, *p* = 0.001). Post hoc analysis indicated that *w*VT_2_ under the Ht-wet condition was significantly lower than under Nt-dry (*p* = 0.001, *d* = 2.26, 95% CI [15.92, 44.08]) and Ht-dry (*p* = 0.027, *d* = 1.45, 95% CI [2.39, 32.62]) conditions. In contrast, no significant difference was observed between Nt-dry and Ht-dry conditions (*p* = 0.096, *d* = 1.04, 95% CI [−2.62, 27.62]).

### 3MT Exercise Performance

**Figure 1:**
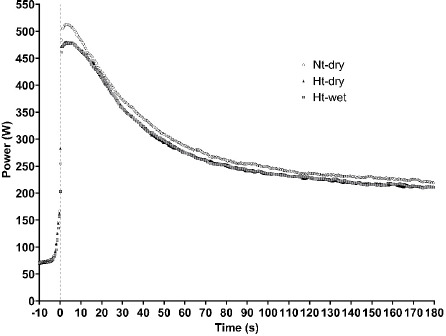
Power-time profiles during the 3MT performed in Nt-dry, Ht-dry, and Ht-wet environments. Note: Ht-wet: a high temperature with high relative humidity condition; Ht-dry: a high temperature with low relative humidity condition; Nt-dry: neutral temperature with low relative humidity

**Figure 2:**
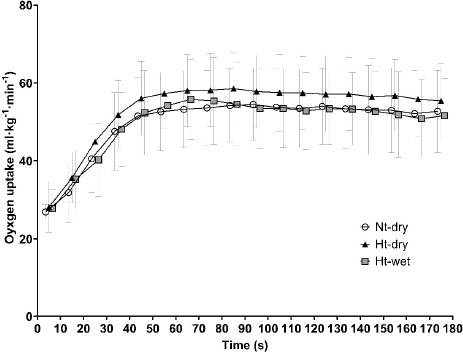
VO_2_ kinetics during the 3MT conducted in Nt-dry, Ht-dry, and Ht-wet environments. Note: Ht-wet: a high temperature with high relative humidity condition; Ht-dry: a high temperature with low relative humidity condition; Nt-dry: neutral temperature with low relative humidity

Figures 1 and 2 illustrate the power-time curves and VO_2_ kinetics, respectively, during the 3MT under different conditions. The effects of environmental conditions on 3MT performance are presented in Table 3. For the calculated 3MT workload derived from *w*VT_1_ and *w*VO_2__max_, a significant main effect of the environmental condition was observed (F(2,22) = 9.46, *p* = 0.001). Post hoc analysis indicated that the workload was significantly higher under the Nt-dry condition compared with Ht-dry (*p* = 0.005, *d* = 0.91, 95% CI [3.45, 14.89]) and Ht-wet (*p* = 0.003, *d* = 1.08, 95% CI [4.65, 17.02]) conditions. In contrast, no significant difference was observed between Ht-dry and Ht-wet conditions (*p* = 0.540, *d* = 0.36, 95% CI [−4.13, 7.47]). For performance indices, significant main effects of the environmental condition were observed for EP (F(2,22) = 4.59, *p* = 0.022), peak power (F(2,22) = 6.20, *p* = 0.007), and average power (F(2,22) = 10.09, *p* = 0.001). Post hoc analysis indicated that EP, peak power, and average power were significantly lower under the Ht-dry condition (EP: *p* = 0.034, *d* = 0.66, 95% CI [0.94, 20.59]; peak power: *p* = 0.008, *d* = 1.37, 95% CI [9.24, 48.42]; average power: *p* = 0.007, *d* = 1.88, 95% CI [4.11, 20.83]) and the Ht-wet condition (EP: *p* = 0.041, *d* = 0.64, 95% CI [0.53, 20.56]; peak power: *p* = 0.001, *d* = 1.51, 95% CI [16.18, 47.32]; average power: *p* = 0.003, *d* = 2.14, 95% CI [5.34, 19.67]) compared with the Nt-dry condition. In contrast, no significant differences were observed between Ht-dry and Ht-wet conditions for EP (*p* = 0.942, *d* = 0.04, 95% CI [−6.78, 6.34]), peak power (*p* = 0.826, *d* = 0.13, 95% CI [−25.67, 31.50]), nor average power (*p* = 0.988, *d* = 0.01, 95% CI [−5.30, 5.38]). WEP did not differ among the three environmental conditions. Regarding physiological responses, no significant differences were observed in VO_2peak_ or HR_peak_ across the environments.

**Table 3:** Effects of environmental temperature and humidity on 3MT performance.

Variables	Nt-dry			Ht-dry			Ht-wet			F	*p*
VO_2peak_ (ml·kg^−1^·min^−1^)	56.9	±	8.7	57.8	±	8.7	58.7	±	8.2	2.110	0.145
3MT load (N)	157.5	±	17.3	148.3	±	13.9^*^	146.7	±	19.5^*^	9.456	0.001
EP (W)	224.7	±	46.7	213.9	±	42.9^*^	214.1	±	44.5^*^	4.590	0.022
WEP (kJ)	11.9	±	2.0	11.5	±	2.8	11.5	±	2.5	0.260	0.774
Peak power (W)	522.5	±	71.6	493.7	±	78.5^*^	490.8	±	72.1^*^	6.203	0.007
Average power (W)	290.5	±	45.2	278.1	±	42.1^*^	278.0	±	45.0^*^	10.094	0.001
HR_peak_ (bpm)	174.3	±	12.4	175.2	±	13.3	176.4	±	11.4	1.663	0.212
RPE (score)											
Baseline	7.3	±	1.1	7.0	±	1.2	7.2	±	1.3	0.348	0.710
Post-exercise	18.8	±	1.1	18.9	±	1.2	19.4	±	0.7	2.554	0.101

Note: Ht-wet: a high temperature with high relative humidity condition; Ht-dry: a high temperature with low relative humidity condition; Nt-dry: neutral temperature with low relative humidity; VO_2peak_: peak oxygen uptake; EP: end power; WEP: anaerobic work capacity; *HR_peak_*: peak heart rate; *RPE*: ratings of perceived exertion; * significantly different from Nt-dry (p < 0.05)

## Discussion

This study primarily examined the effects of high temperature and humidity on EP derived from the 3MT in cyclists. In addition, IET variables, including ventilatory thresholds and submaximal power output, were analyzed to provide complementary information on physiological responses to environmental stress. The main findings were that high ambient temperature significantly impaired EP and other indices of 3MT performance, while VTs and submaximal power output were also reduced, particularly under the Ht-wet condition. In contrast, maximal responses such as VO_2__max_ and HR_max_ remained largely unaffected. These results support the primary focus of the study on EP derived from the 3MT, while the IET variables provide additional insight into submaximal physiological responses to heat stress.

With respect to the IET-derived variables, VO_2max_, *w*VO_2max_, and HR_max_ were not significantly different across conditions. However, submaximal indicators, including VT_1_, VT_2_, *w*VT_1_, and *w*VT_2_, were significantly reduced under high-temperature conditions, especially under the Ht-wet condition. These findings indicate that heat stress accelerates the onset of fatigue during submaximal exercise, which is consistent with previous reports showing that elevated ambient temperature reduces exercise tolerance at moderate intensities without necessarily affecting VO_2max_ (González-Alonso et al., 1999; Périard et al., 2021). The decrease in ventilatory thresholds under Ht-wet conditions may reflect impaired skeletal muscle oxygen delivery and increased cardiovascular strain associated with heat stress, as reported in previous research (Périard et al., 2015).

Exercise time to exhaustion during the IET was shortened under the Ht-dry compared with the Nt-dry condition, supporting the notion that high temperature reduces tolerance to prolonged submaximal exercise (Pilch et al., 2022). Although exercise time to exhaustion under the Ht-wet condition was lower than under the Nt-dry condition, the difference did not reach statistical significance; therefore, the influence of humidity on this variable cannot be confirmed. Previous studies have suggested that hot-humid environments may increase physiological strain and impair heat dissipation (Bright et al., 2025; Williamson-Reisdorph et al., 2023). In addition, performance impairments under high temperature and humidity may reflect a combination of thermoregulatory, cardiovascular, and muscular factors (Périard et al., 2015). Nevertheless, these mechanisms were not directly measured in the present study, and the proposed explanations should therefore be considered as inferences based on previous literature rather than direct physiological evidence.

The 3MT workload, EP, peak power, and average power were all significantly higher under the Nt-dry compared with Ht-dry and Ht-wet conditions, while WEP remained unaffected. These results suggest that maximal effort performance over short duration is sensitive to heat stress, corroborating previous studies reporting reduced critical power under hot conditions (Girard et al., 2015; Kuo et al., 2017). Interestingly, although VO_2peak_ and HR_peak_ did not differ among conditions, EP was impaired under heat stress, highlighting the dissociation between central cardiorespiratory capacity and actual mechanical output in hot environments. This suggests that peripheral factors, such as muscle metabolic efficiency and neuromuscular function, may limit performance in high-temperature conditions despite preserved central capacity (González-Alonso and Calbet, 2003). These include reduced muscle contractility, altered metabolic processes, and increased fatigue, which are exacerbated by heat stress and can occur independently of central limitations (Nybo et al., 2014; Périard et al., 2011).

In the present study, high ambient temperature significantly impaired performance during the 3MT, whereas humidity did not exert an additional measurable effect. This contrasts with the findings for ventilatory thresholds, which were more adversely affected under the Ht-wet condition, suggesting that humidity may exacerbate physiological strain during submaximal exercise. These results indicate that thermal stress per se plays a more critical role than relative humidity in limiting short-duration maximal efforts (Fan et al., 2024; Shi et al., 2022). One possible explanation is that the relatively brief duration of the 3MT may not allow sufficient time for humidity to markedly exacerbate cardiovascular or physiological strain. While humidity is known to impair evaporative heat loss and accelerate thermal load during prolonged endurance exercise (Bright et al., 2025), its impact may be less pronounced in short-term, high-intensity exercise bouts where the primary limitation stems from elevated core and muscle temperature rather than impaired sweat evaporation (Fan et al., 2024; Shi et al., 2022). In contrast, for supra-maximal efforts such as the 3MT, heat-induced decrements in neuromuscular function and metabolic processes may dominate, regardless of ambient humidity. This distinction underscores the importance of differentiating between exercise modalities when interpreting environmental influences on performance.

It should be noted that the workload used for the 3MT in the present study was determined from *w*VT_1_ and *w*VO_2__max_ obtained under each environmental condition. Because ventilatory thresholds and maximal aerobic capacity are themselves influenced by environmental stress, this environment-specific calibration may have affected the absolute workload applied during the 3MT and thus the comparability of EP across conditions. Previous studies have similarly reported that heat stress can influence CP-related variables. Using the conventional multiple constant-load CP method, acute heat exposure at 36°C reduced CP by approximately 6.5% (Bourgois et al., 2023). In contrast, when the 3MT protocol was used under elevated core temperature (~38.5°C), EP was not significantly different between hot (38°C) and thermoneutral (18°C) environments, suggesting that the relatively short duration of the 3MT may limit the expression of heat-induced performance decrements (Kaise et al., 2021). Nevertheless, reductions in key thresholds under heat stress suggest that training prescriptions based on EP obtained in thermoneutral environments may overestimate tolerable intensities in the heat (Kuo et al., 2021). Therefore, EP derived from the 3MT should be interpreted as a CP-like marker within a given thermal environment rather than a directly comparable value across different environmental conditions.

Limitations of this study include the relatively small sample size, which may have limited the statistical power to detect smaller differences between the Ht-dry and Ht-wet conditions, particularly for all-out sprint performance during the 3MT. Future studies with larger sample sizes are warranted to further clarify the impact of humidity on supra-maximal exercise performance in hot environments.

## Conclusions

The primary outcome of this study was EP derived from the 3MT. High ambient temperature impaired ventilatory thresholds and reduced 3MT performance indices (EP, peak power, and average power) despite preserved VO_2__max_ and HR_max_. As secondary outcomes, IET-derived variables showed that high humidity further exacerbated reductions in ventilatory thresholds and threshold-related power outputs during incremental exercise, but did not produce additional decrements in 3MT-derived EP beyond those induced by heat alone. Accordingly, threshold-based training variables appear particularly sensitive to hot-humid stress, whereas short-duration maximal 3MT performance is mainly temperature-dependent in recreational cyclists.
